# Microbial inactivation and shelf life of apple juice treated with high pressure carbon dioxide

**DOI:** 10.1186/1754-1611-3-3

**Published:** 2009-02-04

**Authors:** Giovanna Ferrentino, Mariacarmela Bruno, Giovanna Ferrari, Massimo Poletto, Murat O Balaban

**Affiliations:** 1 Department of Chemical and Food Engineering, University of Salerno via Ponte Don Melillo, 84084 Fisciano (SA) Italy; 2 Centro Regionale di Competenza sulle Produzioni Agroalimentari (PRODAL S.c.a.r.l.) via Ponte Don Melillo, 84084 Fisciano (SA) Italy; 3 Fishery Industrial Technology Center, University of Alaska Fairbanks FITC, 118 Trident Way, Kodiak, AK 99615, USA

## Abstract

Apple juice prepared from 'Annurca' apple puree was treated with a HPCD batch system. The pH, °Brix, color parameters and microbial load of the treated apple juice were compared with those of thermally processed juice. Thermal processes were carried out at 35, 50, 65, 85°C and treatment times ranging between 10 and 140 minutes. Microbial inactivation kinetics indicated that 5-log reduction of natural flora in apple juice was achieved at 85°C and 60 minutes of treatment time for conventional thermal process and at 16.0 MPa, 60°C and 40 minutes for HPCD process. Results suggested that temperature played a fundamental role on HPCD treatment efficiency, with inactivation significantly enhanced when it increased from 35 to 60°C. Less significant was the role of the pressure at the tested levels of 7.0, 13.0 and 16.0 MPa. Also, 5-log reduction of natural flora in apple juice was obtained at lower temperatures by cyclic treatments of six compression and decompression steps. There were no significant differences between treated and untreated samples in °Brix (α = 0.05). Significant differences were detected in pH values between the untreated and HPCD treated samples (α = 0.05). There was a significant decrease in 'L*' and 'b*' values and also differences were detected in 'a*' values between the untreated and the HPCD treated samples (α = 0.05). Statistical analysis for °Brix, pH and color data showed no differences between the untreated and HPCD treated samples in the first 2 weeks of storage at 4°C. These results emphasize the potential use of HPCD in industrial applications.

## Introduction

The consumer demands for safe and minimally processed food with high quality attributes have encouraged the food industry to find innovative processes. Frequently investigated non-thermal microbial inactivation technologies are high hydrostatic pressure (HHP), pulsed electric fields (PEF), new packaging systems such as modified atmosphere packaging (MAP) and active packaging, natural antimicrobial compounds and bio-preservation [1]. Currently there is a growing interest in high pressure carbon dioxide (HPCD) as an alternative processing method and studies using its combination with mild temperatures for pasteurization of liquid food have been performed. This method is attracting interest in the food industry because microbial inactivation is achieved, and no taste, or aroma changes are perceived and vitamin quality is maintained.

Many authors have reported experimental evidence of the effects of HPCD on different substrates and different microorganisms commonly present in foods, both in their vegetative and spore forms [2-5]. Recently several experimental works and reviews [6,7] have been published regarding the effect of CO_2 _under pressure and its mechanisms of inactivation on vegetative microorganisms, spores and enzymes. Also a study about the influence of compression and decompression rate (besides temperature, pressure, exposure time, water content and initial pH) on the physiology of *Absidia coerula *and *Saccharomyces cerevisiae *has been performed by Liu et al. (2005) [8].

Kincal et al. (2005) [9] treated orange juice with HPCD showing a 5-log reduction in microbial load while maintaining the chemical and sensory qualities of the untreated product. Little differences were detected in °Brix, pH and color, while titratable acidity increased slightly for the treated samples compared with the untreated. Spilimbergo and Mantoan (2006) [10] applied HPCD treatment to an apple juice, achieving total microbial inactivation at 10.0 MPa, 36°C, 5 mL sample volume and with 1 minute of treatment time in a multi – batch apparatus. Further experiments were performed on cloudy apple juice to avoid enzymatic browning caused by the action of polyphenol oxidases. The investigations showed that the treatment had significant effects, and the polyphenol oxidases had more than 60% activity reduction at 30 MPa and 55°C for 60 minutes. The color analysis, performed on the cloudy apple juice, showed a decrease of lightness, a minor increase of redness and no changes in yellowness [11].

Studies were also carried out to show the effect of the decompression rate on microbial cell viability. Fraser (1951) [12] was among the first to demonstrate disruption of bacterial cells by the rapid release of gas pressure with the aim of collecting cell contents. More recently, Liu et al. (2005) [8] concluded that the viability and biological activity of *S. cerevisiae *and also of spores of *Absidia coerulea *was significantly influenced by the CO_2 _decompression rate (7.5 MPa, 30°C, 30 and 90 minutes). This evidence was clearly shown for the *A. coerulea *spores which possessed a thicker outer layer as demonstrated by images carried from electron microscopy. Enomoto et al. (1997) [13] exposed *S. cerevisiae *cells to pressurized CO_2 _(4.0 MPa, 40°C, 240 minutes) and then decompressed to atmospheric pressure using two different decompression rates. Their results showed that a rapid or multiple decompressions would not always lead to an increased inactivation, suggesting that the microbial cells might not be ruptured mainly by an explosive decompression. In conclusion, it seems that under certain treatment conditions, cell rupture is possible by HPCD.

Most commercialization efforts have been from Praxair Inc. (Burr Ridge, IL, US). Based on the technology licensed from the University of Florida [14,15], Praxair developed a continuous system which uses HPCD as a non-thermal alternative process to thermal pasteurization [16]. This system has been commercialized under the Trade Mark "Better Than Fresh (BTF)". Praxair has constructed four mobile BTF units for processing about 1.5 liter per minute of juice for demonstration purposes. In addition, a commercial scale unit of 150 liter per minute was also constructed [16] and tested at an orange juice processing plant in Florida. The excellent taste of the juice processed with this new technology was demonstrated in three independent sensory panels that compared juice treated with this system to that of fresh squeezed juice. In all the tests no difference could be detected.

The purpose of this study was to investigate the effect of the HPCD treatments on the microbial inactivation and the changes in color, °Brix and pH of the 'Annurca' apple juice. These parameters were compared with that of the thermally treated and untreated juices. Pressure, temperature and time were the process variables tested in the HPCD batch apparatus. Also effects of compression and decompression cycle experiments on microbial inactivation were studied.

## Materials and methods

### High Pressure CO_2 _apparatus

**Figure 1:**
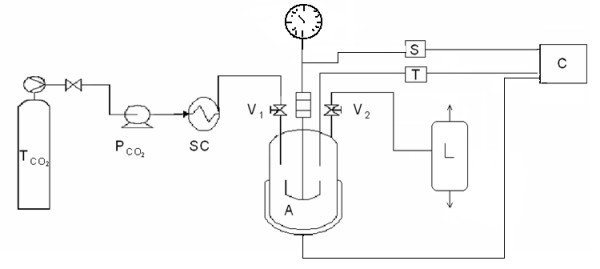
**The experimental apparatus: A, vessel with external heater; C, Temperature and magnetic stirrer PID controller; T, thermocouple; S, magnetic stirrer; L, liquid separator**. P_CO2_, carbon dioxide pump; SC, cooler system with Peltier effect; T_CO2_, carbon dioxide tank; V_1_, inlet globe valve; V_2_, outlet globe valve.

The experimental apparatus was a batch reactor (Figure 1), that allowed the treatment of liquid and solid food with CO_2 _under pressure. The vessel (Parr Stirred Reactor, FKV srl, Bergamo, Italy) had 100 mL of volume, with a maximum operating pressure of 20.0 MPa. A four-bladed impeller magnetically coupled to a DC motor (model No. A1120HC6) allowed adjustable mixing speed, and a fixed thermocouple (Type J) was used for temperature measurement. The vessel head could be removed to clean the system and to introduce liquid samples for batch experiments. It was equipped with a calibrated rupture disc for emergency pressure relief. The vessel bottom was fitted in a circular electric heater connected to an A-4840 Series PID Temperature Controller, calibrated to rapidly bring the system to the desired temperature with a minimum overshoot and continuous regulation. A pressure gauge, fitted on the reactor head, displayed the pressure inside the system.

The liquid CO_2 _was pumped by a volume displacement pump (Jasco, model PU-1580) in which the fluid flow could be controlled either for constant volume or for constant pressure throughput, up to a maximum output pressure of 30.0 MPa. The liquid CO_2 _(99.99% purity, SOL SpA Italy) was pumped into the reactor through an on-off valve that was kept closed after pumping for the time required for the experiments. The time required for the pressurization of the reactor and for the heating was about 5 minutes. The depressurization step occurred by manually opening the on – off valve on the vessel outlet line. During this step the temperature decreased of about 3°C inside the system. The sample, loaded in the reactor, was collected after the depressurization of the system. In all the tests the same stirring speed (approx. 850 rpm) was used.

### Apple juice

'Annurca' apple fruit, which is commonly cultivated in Southern Italy, is known for its firmness. It is an old apple cultivar commonly grown in the Campania region. It accounts for 95% of Southern Italy production and 3 – 4% of national apple production [17].

After harvest, apples were washed and manually sliced to increase the surface and to improve the absorbance of citric and ascorbic acid in the following steps. The slices were immediately immersed in a solution of ascorbic and citric acids, (both 0.2% w/w) to slow down the browning process. The slices were then processed in a crusher, and exposed to a direct steam injection. The softened slices were torn easily by the action of rotating beaters. Finally the product was mechanically reduced to puree, cooled with ice and filled in bottles. The bottles were stored at + 4°C before further treatments. The juice was obtained by mixing the puree with fructose and water to correct the acidic taste. The final composition of the juice was: 50 g of apple puree and 7.5 g of fructose in 50 g of water. The untreated juice was frozen immediately after production to preserve its original quality characteristics and thawed throughout the experiments as a baseline; treated juice was thawed and exposed to various HPCD treatments and storage conditions; and the thermally treated juice was the untreated juice exposed to a thermal treatment.

### Growth media

To detect the total aerobic bacterial growth of the natural apple juice, a non – selective culture media PCA plate count agar (composition: pancreatic digest of casein 5.0 g/L, yeast extract 2.5 g/L, dextrose 1.0 g/L, agar 15.0 g/L) was used. The initial microbial count of the microorganisms in the apple juice obtained from the puree was in the range 10^5 ^– 10^6 ^colony forming unit per mL (cfu/mL). The final count of the microorganisms, grown on PCA agar slant on Petri dishes, took into account the multiplicative factors depending on the inoculation volume and dilution. The final concentration was obtained by the arithmetic mean of different counts on the same sample. Each experiment was run three times and the arithmetic mean was reported as the final result. For each test the survival fraction S = N/N_0 _was determined, where N_0 _was the number of microorganisms initially contained in the sample (control sample) and N was the corresponding number after the treatments. From the survival fraction, the level of inactivation Log(S) was evaluated.

### Thermal inactivation

Apple juice (50 mL) was placed in the vessel and heated to the desired temperature and time without the addition of CO_2 _under pressure. To examine the effect of temperature during the pasteurization, the treatments were carried out at 35, 50, 60, and 85°C for 10, 20, 30, 40, 60, 80 and 120 minutes. The heating time was recorded after the juice reached the desired temperatures. The time required for the system to reach the set temperature was about 5 minutes. After the treatment, the samples were collected in 100 mL sterile bottles, cooled in an ice bath for about 10 minutes and then analyzed.

### High pressure carbon dioxide inactivation

Experiments were carried out with HPCD at the same temperatures used for the thermal treatments. Temperatures higher than 60°C were not considered because at these temperatures the advantages of using the mild conditions of HPCD were lost. For each experiment, 50 mL of apple juice was placed in the vessel, heated to the experimental temperature and then pressurized by CO_2_. The increase in the temperature in the vessel was about 2 – 3°C during pressure build-up and returned to the experimental level when the pressure reached the experimental level. The sample was held at constant temperature and pressure during the treatment time. At the end, the vessel was slowly depressurized causing a decrease in temperature of about 4 – 5°C. After the treatment, the sample was removed, placed in 100 mL sterile bottle, cooled in an ice bath for about 10 minutes and then analyzed.

### Compression and decompression treatment

To better understand the effect of compression and decompression cycles on the inactivation of microorganisms, 50 mL samples were placed in the vessel, heated to 35°C and then pressurized by CO_2 _to 13.0 MPa. Six compression and decompression cycles were performed. Each cycle was carried out at the same conditions for 10 minutes of treatment time. After each cycle, the apple juice was removed, placed in 100 mL sterile bottle, cooled in an ice bath for about 10 minutes and the microbial load, °Brix, pH and color were measured when complete inactivation was achieved.

### Storage study

The apple juice treated at 16.0 MPa, 60°C and 40 minutes of treatment time, and the apple juice thermally treated at 85°C for 60 minutes were compared with the untreated juice. A storage study was also performed on the apple juice after the sixth compression and decompression cycle. The untreated, thermally, HPCD, and compression and decompression treated juices were stored at 4°C and aliquots were taken and analyzed by the methods described below on a weekly basis.

### pH determination

The pH of HPCD treated, untreated and thermally treated apple juice samples was measured using a digital pH meter (Seven Easy, Mettler Toledo, Inc. Columbus, OH), calibrated with commercial buffer solutions at pH 7.0 and 4.0. A sample (20 mL) was placed in a 50 mL beaker with a magnetic stirring bar and pH electrode inserted. The pH was recorded after stabilization at ambient temperature.

### °Brix determination

A digital refractometer with temperature correction (Abbe, DR-A1 Houston, TX) was used. A representative sample from juices was taken by a disposable plastic pipette and °Brix values were determined. The refractometer prism was cleaned with distilled water after each analysis. Accuracy of the refractometer was checked by determining the refractive index for distilled water.

### Color analysis

Color was measured using a Chroma Meter CR-200b (Konica Minolta Business Solutions, Milano Italia). It was able to diffuse a ray of light, generated by a pulsed electric arch. The observation angle was 0° compared to the surface of interest. The ray of light reflected in every direction in the chamber, designed to contain the liquid, and hit the surface of the sample. The ray reflected perpendicularly to the surface of the sample for the measurement of its color. It was captured by an optical fibre and was divided in three components through special filters, for characterization. The results were expressed in the CIE system (1931). For calibration a white reference standard plate was used. The standard parameters were recorded. The sample cup was fitted onto the circular sample port within the illumination area. The color parameters of the sample were displayed in terms of 'L*' (lightness), 'a*' (redness), 'b*' (yellowness) values. Changes in color were expressed as:

(1) $$\Delta \text{E}=\sqrt{{\left({\text{L}}_{\text{before}}^{*}-{\text{L}}_{\text{after}}^{*}\right)}^{\text{2}}+{\left({\text{a}}_{\text{before}}^{*}-{\text{a}}_{\text{after}}^{*}\right)}^{\text{2}}+{\left({\text{b}}_{\text{before}}^{*}-{\text{b}}_{\text{after}}^{*}\right)}^{\text{2}}}$$

### Statistical analysis

For chemical analyses, differences between the untreated, the thermal, HPCD and the compression and decompression treated samples were analyzed using one way ANOVA (Statistica Software 7.0; Statsoft, Tulsa, OK). The data were statistically analyzed with the Tukey HSD test for means comparison (α = 0.05).

## Results and Discussion

### Inactivation due to thermal treatment

The results showed that it was necessary to increase the temperature up to 85°C with a treatment time of 80 minutes to achieve a 5-log microbial inactivation. In particular at 60°C and lower it was not possible to reduce microbial populations more than 3-log cycles with a treatment time of 120 minutes.

### Inactivation due to combined thermal and HPCD treatment

**Figure 2:**
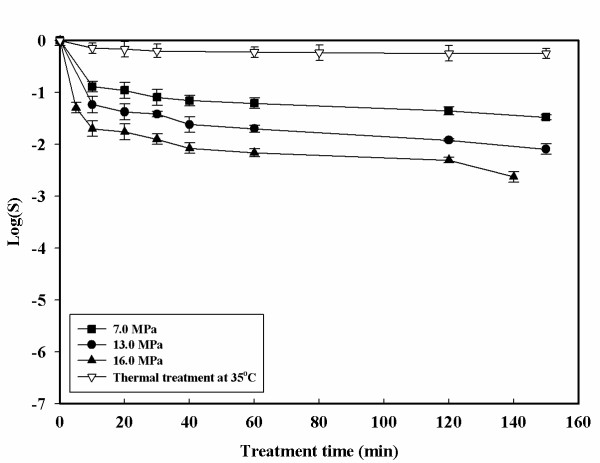
**Microbial inactivation of apple juice treated with HPCD as a function of the treatment time for different pressure at 35°C**. Values are means of 3 processing runs.

**Figure 3:**
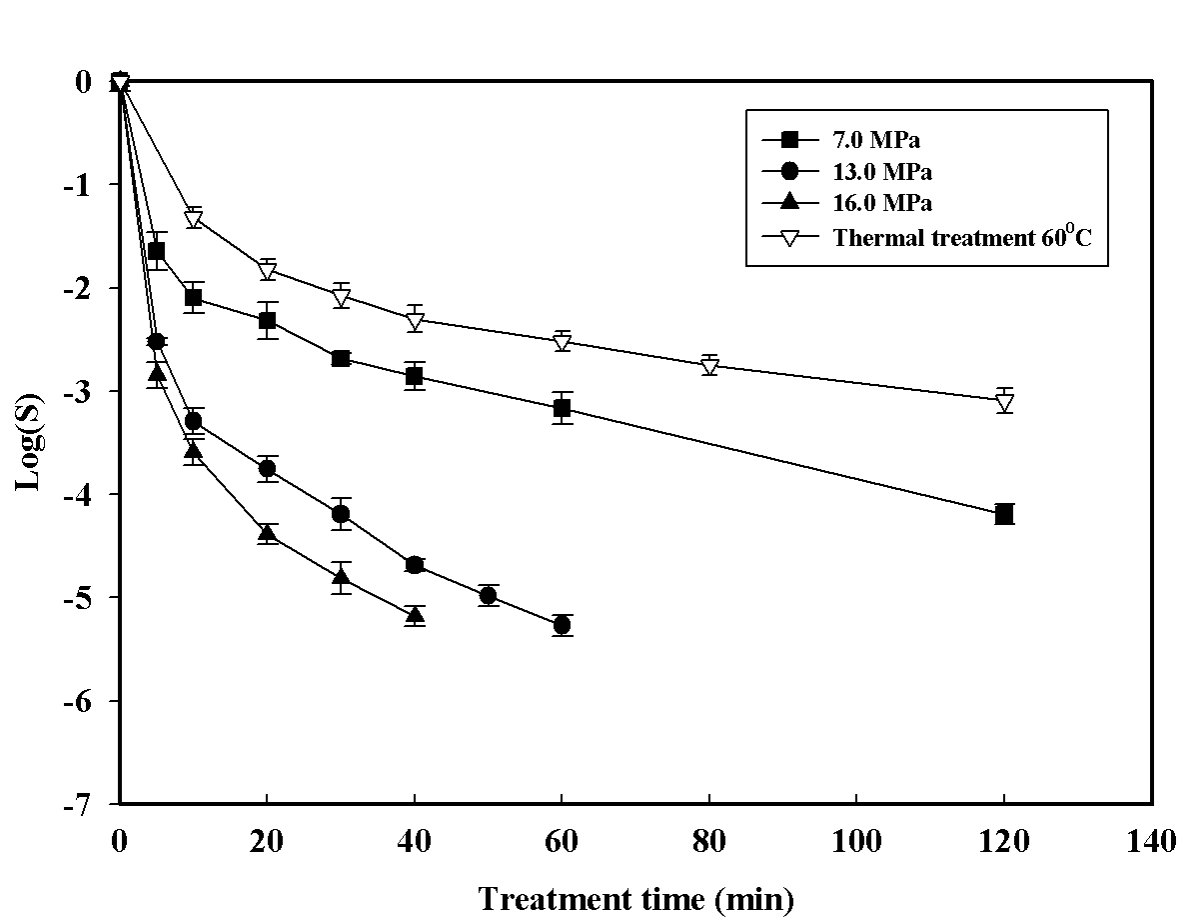
**Microbial inactivation of apple juice treated with HPCD as a function of the treatment time for different pressures at 60°C**. Values are means of 3 processing runs.

**Figure 4:**
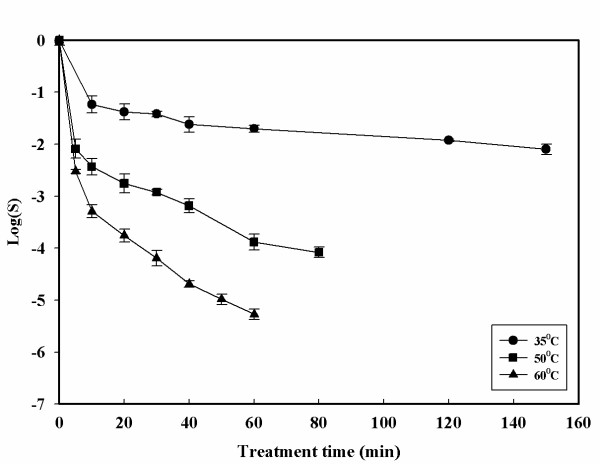
**Microbial inactivation of apple juice treated with HPCD as a function of the treatment time for different temperatures at 13.0 MPa**. Values are means of 3 processing runs.

**Figure 5:**
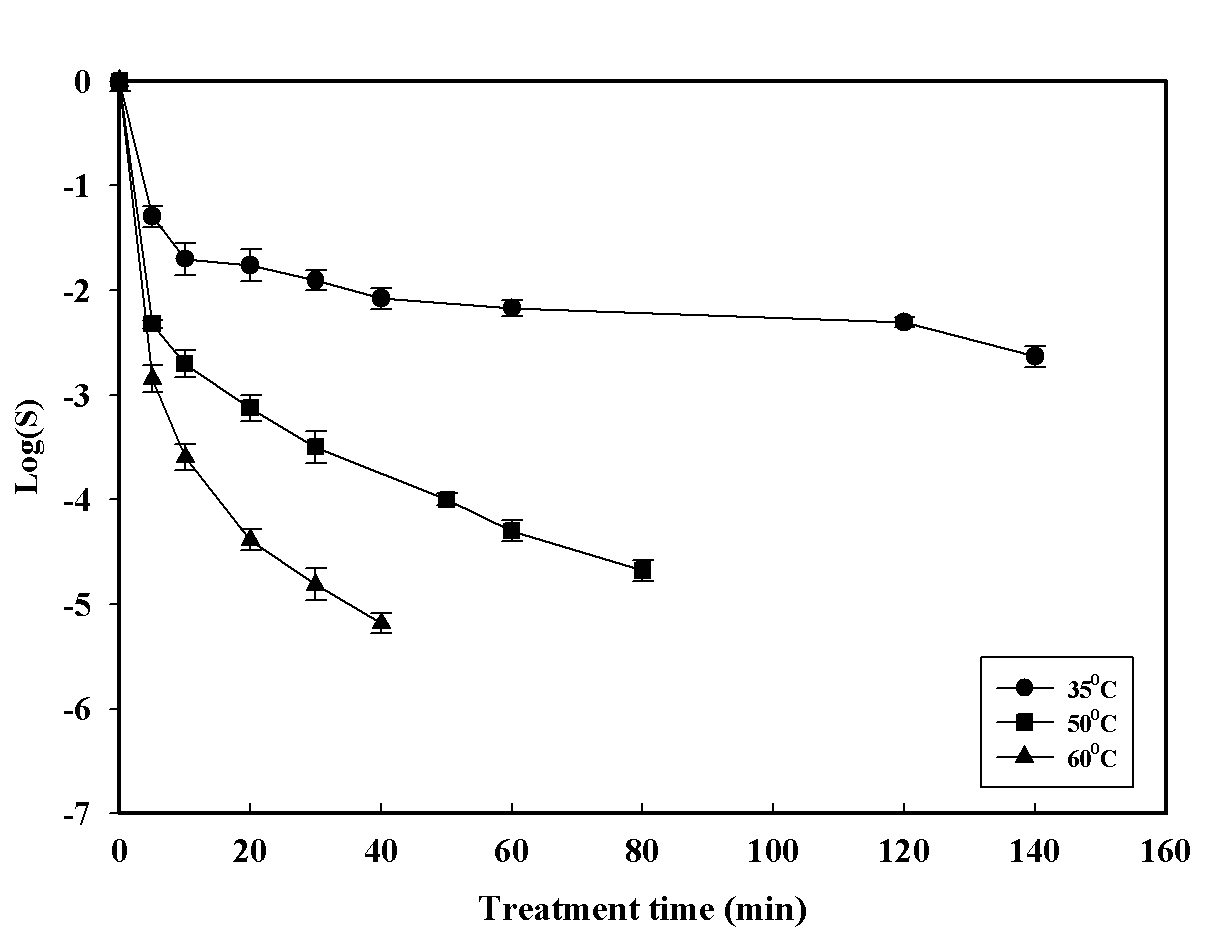
**Microbial inactivation of apple juice treated with HPCD as a function of the treatment time for different temperatures at 16.0 MPa**. Values are means of 3 processing runs.

Inactivation curves with the HPCD treatment are shown in Figures 2, 3, 4, 5. The inactivation kinetics is clearly non – linear and cannot be described by a first order model. In Figure 2 at 35°C a very slight inactivating action of the treatments is reported. An increase in pressure from 7.0 to 16.0 MPa does not show a large increase in the inactivation values. Increasing the pressure at this temperature it is possible to achieved only 2.5-log reduction after 140 minutes. Figure 3 shows the effects of pressure at 60°C compared to a thermal treatment at the same temperature. Changing the temperature from 35 to 60°C resulted in faster inactivation rates. At 16.0 MPa and 60°C, 5-log reduction is obtained after 40 minutes of treatment time. The pressure controlled both the solubilization rate of CO_2 _and its solubility in a suspending medium. Consequently, higher pressure enhanced CO_2 _solubilization to facilitate its contact with the cells. However, the observed differences in microbial inactivation between 7.0 and 16.0 MPa at fixed temperature cannot be simply attributed to the changes in CO_2 _solubility. Not only pressure but also temperature is closely related to CO_2 _mass transfer. Higher temperatures increase the diffusivity of CO_2 _and can also increase the fluidity of cell membrane to make its penetration easier. Figures 4 and 5 show the effect of the temperature at 13.0 and 16.0 MPa. Under constant CO_2 _pressures of 13.0 and 16.0 MPa, the inactivation rate increased with increasing temperature. At 35°C and 7.0 MPa (Figure 2) insignificant inactivation is achieved even increasing the treatment time to 120 minutes, while at the same pressure but at 60°C after 120 minutes of treatment time a 4-log reduction is achieved. In contrast to what was observed with simple thermal treatment, within this range the effects of temperature start to become noticeable. Figure 5 also shows that an increase in temperature up to 60°C can also reduce the treatment time. Similar trends but more effective results of HPCD were found by Spilimbergo and Mantoan (2006) [10] treating apple juice in a multi – batch system. They achieved total microbial inactivation at particularly mild conditions: 10.0 MPa, 36°C, 1 minute of treatment time and with 5 mL of sample volume. They observed that sample volume was a crucial parameter in the inactivation process if the pasteurization was carried out in a batch system. In this work experiments were carried out with 50 mL of sample volume. This could be a possible explanation to the high pressure and temperature conditions needed to achieve a 5-log microbial inactivation. They also showed that the temperature played an important role in the treatment efficiency. The inactivation was enhanced by increasing the temperature in the range of 25 – 36°C.

### Inactivation by compression and decompression treatment

**Figure 6:**
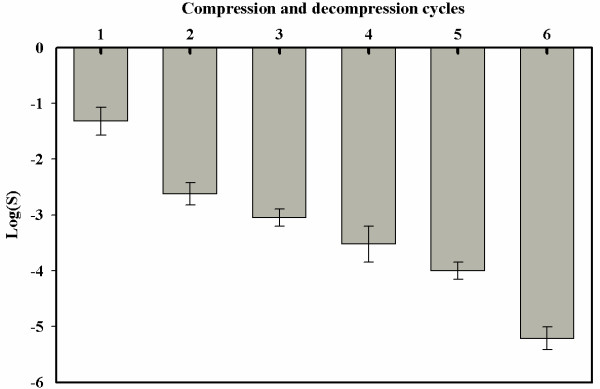
**Microbial inactivation of apple juice for six compression and decompression cycles**. Each cycle performed at 13.0 MPa, 35°C for 10 minutes. Values are means of 3 processing runs.

Figure 6 shows the microbial inactivation of six compression and decompression cycles. After the sixth cycle 5-log microbial reduction was achieved. These results agreed with what was found in other studies [12,18-22]. The compression and decompression treatment consists of a pressurization stage to first induce penetration of CO_2 _into the microbial cells, followed by a sudden release of pressure from the suspending medium that results in rapid gas expansion within the cells. There are two theories on how a pressure cycling operation can enhance deactivation: an enhanced cell rupture theory [12] and an enhanced mass transfer theory [23-25]. Fraser (1951) [12] achieved more than 90% cell rupture only after two pressure cycles, at least a 15% increase from a single pressurization step. Dillow et al., (1999) [23] also observed that with a treatment at 34°C, 20.5 MPa and total treatment time 0.6 h, the degree of inactivation jumped from 3-log with three pressure cycles to 9-log with six pressure cycles.

### °Brix, pH and color

Treatments with batch HPCD did not cause any change in °Brix levels of apple juice (Table 1). There were no significant differences in °Brix values between HPCD treated, untreated and thermally treated samples (α = 0.05). Similar results were observed by Arreola et al. (1991) [26] and Kincal et al. (2005) [9] when orange juice was treated with a static supercritical CO_2 _and with a continuous high pressure CO_2 _system, respectively. Significant changes are detected in pH values comparing the untreated and the thermally treated samples with the HPCD treated samples (α = 0.05). The differences were mainly due to some of the dissolved CO_2 _remaining in solution after pressure is released from the system. In commercial practice, the residual CO_2 _is removed by "de-gasification" of the juice.

**Table 1:** Apple juice pH and °Brix values before and after the treatments

Pressure (MPa)	T (°C)	Time (min)	Untreated^a ^pH	Treated^a ^pH	Untreated^a ^°Brix	Treated^a ^°Brix
HPCD treatments						

7.0	35	150	3.56^b^ 14.70^c^	3.52^c^	14.70^f^	14.70^f^
13.0	35	150	3.55^b^ 14.71^c^	3.50^c^	14.71^f^	14.72^f^
13.0	50	80	3.56^b^ 14.71^c^	3.50^c^	14.71^f^ 14.70^c^	14.70^f^
16.0	50	80	3.55^b^ 14.71^c^	3.51^c^	14.71^f^ 14.72^c^	14.72^f^
16.0	60	40	3.56^b^ 14.72^c^	3.52^c^	14.72^f^ 14.72^c^	14.72^f^

Thermal treatment						

-	85	60	3.57^b^	3.57^d^	14.70^f^	14.70^f^

After the sixth compression and decompression cycle						

13.0	35	10	3.56^b^	3.48^e^	14.70^f^	14.70^f^

^a^Values are means and represent the trends seen for 3 processing runs.

The same superscripts in a column are not significantly different (p < 0.05).

Table 2 shows the color parameters for the untreated, thermally treated and HPCD treated samples. The 'L*' (lightness) value shows significant differences between the untreated and the HPCD treated samples and the 'a*' (redness) value of HPCD apple juices display slight decrease when compared to the untreated samples (α = 0.05). The 'b*' (yellowness) value shows significant differences between the untreated, the compression and decompression and the HPCD treated sample. Also significant differences are detected in the "b*" values between the HPCD treatment conditions. Increasing the temperature from 35 to 60°C determine a significant decrease of the yellowness of the juice (α = 0.05). Considering the ΔE parameters in Table 2, it is possible to notice that the overall change in color of the juice is not significant different considering the high pressure and temperature tested. Similar observations are reported by Jwa et al. (1996) [27] showing that supercritical CO_2 _treatment rises the 'L*' value of citrus fruit juice. Park et al. (2002) [28] found that a combined treatment of high hydrostatic pressure and CO_2 _caused an insignificant increment in the 'L*' value of carrot juice. Furthermore, the difference of 'L*', 'a*' and 'b*' values of all treated samples under various pressures were insignificant immediately after treatment. Fenqi et al. (2006) [11] found that the 'L*' value of cloudy apple juice displayed insignificant increases while its 'a*' and 'b*' values remained almost unchanged immediately after supercritical CO_2 _treatment.

**Table 2:** Apple juice color values before and after the treatments^a^

Pressure (MPa)	T (°C)	Time (min)	L* _Untreated_	L* _Treated_	a* _Untreated_	a* _Treated_	b* _Untreated_	b* _Treated_	ΔE
HPCD treatments									

7.0	35	150	49.17^b^ -0.87^c^ 2.77^c^	48.03^c^	-0.87^d^	-0.83^f^ -0.83^c^ 2.93^c^ 1.15^e^	2.77^g^	2.93^h^	1.15^l^
13.0	35	150	49.50^b^ -0.80^c^ 2.77^c^	48.70^d^	-0.87^d^	-0.80^e, f^ 2.87^c^ 0.83^e^	2.77^g^	2.87^h^	0.83^l^
13.0	50	80	49.73^b^ -0.87^c^ 2.73^c^	48.53^d^	-0.87^d^	-0.87^e, f^	2.73^g^	2.47^i^	1.24^l^
16.0	50	80	49.17^b^ -0.97^c^ 2.77^c^	48.77^d^	-0.97^d^	-0.83^e, f^	2.77^g^	2.33^i^	0.62^l^
16.0	60	40	49.17^b^ -0.97^c^ 2.77^c^	48.67^d^	-0.97^d^	-0.87^e, f^	2.77^g^	2.33^i^	0.68^l^

Thermal treatment									

-	85	60	49.5^b^	48.10^c^	-0.87^d^	-0.90^e^	2.77^g^	2.47^i^	1.44^l^

Sixth compression and decompression cycle									

13.0	35	10	49.17^b^	48.57^d^	-0.93^d^	-0.87^e, f^ -0.87^bc^ 2.83^bc^ 0.62^be^	2.73^g^	2.83^h^	0.62^l^

^a^Values are means and represent the trends seen for 3 processing runs.

The same superscripts in a column are not significantly different (p < 0.05).

### Storage study

**Figure 7:**
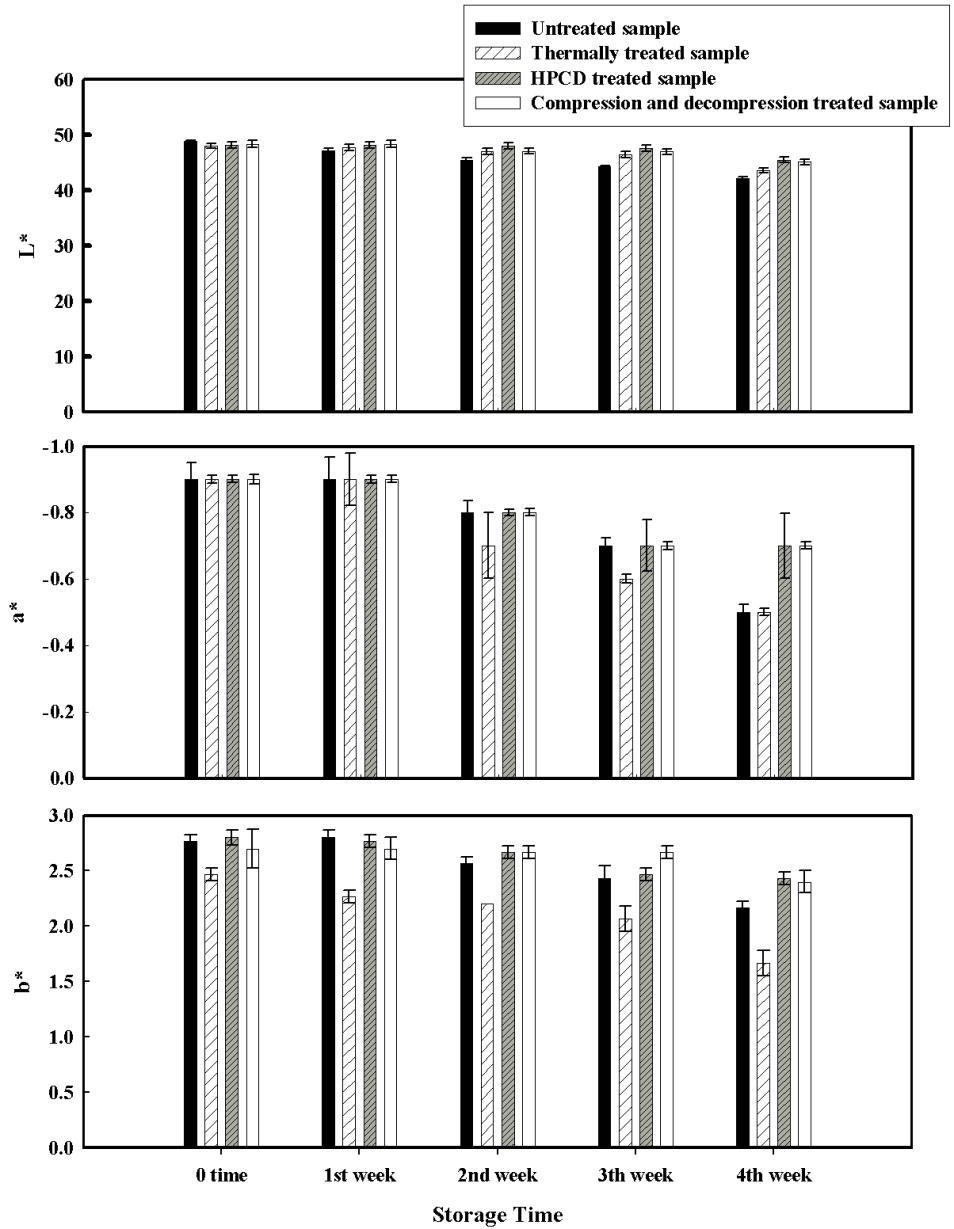
'L*', 'a*' and 'b*' values for untreated, thermally treated, HPCD treated and compression/decompression treated samples as a function of the storage time. The apple juice was treated with heat at 85°C for 60 minutes, with a batch HPCD at 16.0 MPa, 60°C for 50 minutes and with six compression and decompression cycles at 13.0 MPa, 35°C for 10 minutes each.

**Figure 8:**
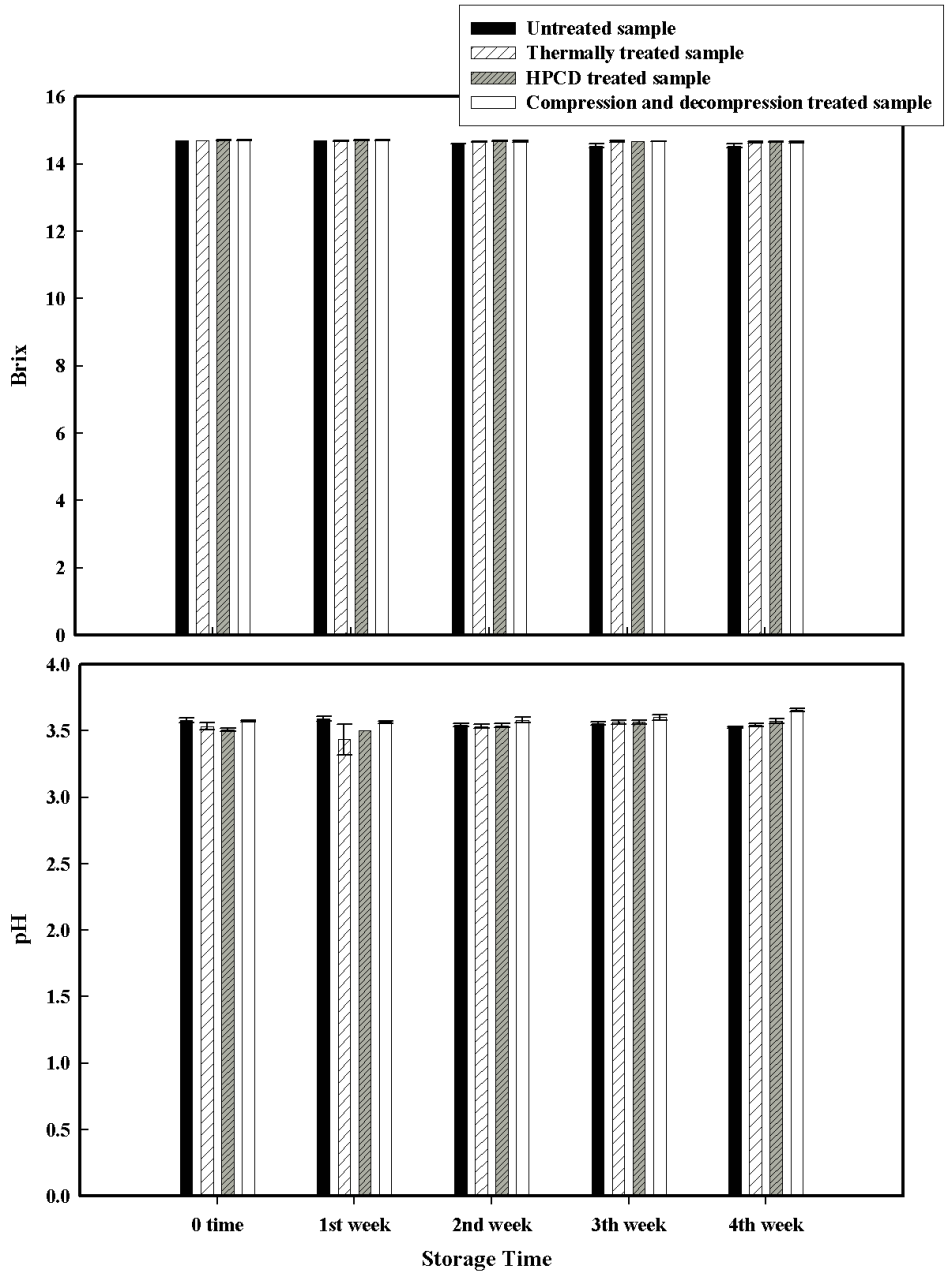
**Brix and pH values for untreated, thermal treated, HPCD treated and compression/decompression treated samples as a function of the storage time**. The apple juice was thermally treated at 85°C for 60 minutes, with a batch HPCD at 16.0 MPa, 60°C for 50 minutes, and with six compression and decompression cycles at 13.0 MPa, 35°C for 10 minutes each.

Figures 7 and 8 report results of the °Brix, pH and color measurement of the stored samples. There was a slight difference between the thermal, HPCD treated and the untreated samples for °Brix and pH values regardless of storage time (Figure 8). Color for stored juice did not change drastically during storage of the HPCD and the compression and decompression treated juices. Figure 7 shows that HPCD samples and the compression and decompression samples retained their initial lightness ('L*') during the first two weeks of storage while the values change significantly during the third and forth weeks (α = 0.05). The untreated sample and the thermally treated sample show a consistent lightness decrease that is already detected after the first week of storage. Inspection of the redness values at different storage times indicates that all the HPCD treated samples have no changes during the first 2 weeks of storage. Juices treated with HPCD have a decrease in redness ('a*') during the last 2 weeks of storage but the untreated sample and the thermally treated sample show a more significant decrease in this value during the same time (α = 0.05). Yellowness ('b*') values for thermal and untreated samples decrease consistently during the storage while for HPCD treated samples it does not change significantly. Similar results are indicated by Fenqi et al. (2006) [11], who observed significant reduction in lightness and redness for untreated samples of cloudy apple juice while the samples treated with supercritical CO_2 _seemed to have smaller changes when compared to the untreated and heat treated samples. Also in their study, the yellowness values of all the samples did not shift significantly during storage. Kincal et al. (2005) [9] showed that orange juice treated with a continuous HPCD system had higher lightness and higher yellowness values during storage when compared with the untreated samples. Microbial load was also checked during the refrigerated storage of the treated juices. No increase in microbial numbers was observed.

## Conclusion

The total aerobic bacterial growth of the apple juice was inactivated by a HPCD treatment carrying out experiments with a batch apparatus changing pressure, temperature and treatment time and considering the effects of these process variables on microbial inactivation and physical attributes of the juice. Various log decreases were observed depending on the pressure, temperature and treatment time conditions. A temperature of 35°C was not able to induce a relevant log reduction even when increasing the pressure to 16.0 MPa. The storage study showed that the treated samples maintained the physical qualities of the untreated juice better than the thermally treated samples. Juice samples treated by these methods showed little differences in Brix, pH and color when compared to the untreated samples. On the basis of these conclusions, the HPCD treatment proved to be a promising alternative technique in the beverage industry to produce juices having fresh like characteristics while extending shelf life and safety.

## Competing interests

The authors declare that they have no competing interests.

## Authors' contributions

GF participated in the design of the experimental procedures, carried out the experiments with the HPCD equipment, performed the statistical analysis and drafted the manuscript. MCB performed the microbiological and shelf life procedures. GF and MP coordinated the study, participated in its design and together with MOB helped to draft the manuscript. All authors read and approved the final manuscript.
